# Timing of Symptoms of Early-Onset Sepsis after Intrapartum Antibiotic Prophylaxis: Can It Inform the Neonatal Management?

**DOI:** 10.3390/pathogens12040588

**Published:** 2023-04-13

**Authors:** Alberto Berardi, Viola Trevisani, Antonella Di Caprio, Paola Caccamo, Giuseppe Latorre, Sabrina Loprieno, Alessandra Foglianese, Nicola Laforgia, Barbara Perrone, Giangiacomo Nicolini, Matilde Ciccia, Maria Grazia Capretti, Chiara Giugno, Vittoria Rizzo, Daniele Merazzi, Silvia Fanaro, Lucia Taurino, Rita Maria Pulvirenti, Silvia Orlandini, Cinzia Auriti, Cristina Haass, Laura Ligi, Giulia Vellani, Chryssoula Tzialla, Cristina Tuoni, Daniele Santori, Lorenza Baroni, Mariachiara China, Jenny Bua, Federica Visintini, Lidia Decembrino, Roberta Creti, Francesca Miselli, Luca Bedetti, Licia Lugli

**Affiliations:** 1 Neonatal Intensive Care Unit, University Hospital of Modena, 41224 Modena, Italy; 2 School of Pediatrics Residency, University of Modena and Reggio Emilia, 41224 Modena, Italy; 3 Neonatal Intensive Care Unit, Ecclesiastical General Hospital F. Miulli, 70021 Acquaviva delle Fonti, Italy; 4 Department of Biomedical Science and Human Oncology (DIMO), Neonatal Intensive Care Unit, University Hospital of Bari “Aldo Moro”, 70124 Bari, Italy; 5 Neonatal Intensive Care Unit, Azienda Ospedaliero Universitaria Ospedali Riuniti, 60126 Ancona, Italy; 6 Pediatric Unit, San Martino Hospital, 32100 Belluno, Italy; 7 Neonatal Intensive Care Unit, Women’s and Children’s Health Department, Maggiore Hospital, 40133 Bologna, Italy; 8 Neonatal Intensive Care Unit, Women’s and Children’s Health Department, S. Orsola-Malpighi Hospital, 40138 Bologna, Italy; 9 Pediatric Unit, Ospedale B. Ramazzini, 41012 Carpi, Italy; 10 Neonatal Intensive Care Unit, Bufalini Hospital, Cesena, 47521 Cesena, Italy; 11 Division of Neonatology, “Valduce” Hospital, 22100 Como, Italy; 12 Department of Medical Sciences, Pediatric Section, University Hospital, 44124 Ferrara, Italy; 13 Neonatal Intensive Care Unit, Ospedali Riuniti, 71122 Foggia, Italy; 14 Pediatric and Neonatal Unit, Morgagni-Pierantoni Hospital of Forlì, 47121 Forlì, Italy; 15 Neonatal Intensive Care Unit, Carlo Poma Hospital, 46100 Mantova, Italy; 16 Neonatal Intensive Care Unit, Medical and Surgical Department of Fetus-Newborn-Infant, “Bambino Gesù” Children’s Hospital IRCCS, 00165 Rome, Italy; 17 Neonatal Intensive Unit, San Pietro-Fatebenefratelli Hospital, 00168 Rome, Italy; 18 Neonatal Intensive Unit, San Filippo Neri Hospital, 00135 Rome, Italy; 19 Neonatal Intensive Unit, ARNAS Civico-Di Cristina-Benfratelli, 90127 Palermo, Italy; 20 Neonatal and Pediatric Unit, Polo Ospedaliero Oltrepò, ASST Pavia, 27100 Pavia, Italy; 21 Neonatology and Neonatal Intensive Care Unit, Department of Clinical and Experimental Medicine, University Hospital of Pisa, 56124 Pisa, Italy; 22 Pediatric and Neonatal Unit, Azienda Ospedaliera Santa Maria degli Angeli, 33170 Pordenone, Italy; 23 Neonatal Intensive Care Unit, Santa Maria Nuova Hospital, 42123 Reggio Emilia, Italy; 24 Neonatal Intensive Care Unit, Infermi Hospital, 47923 Rimini, Italy; 25 Neonatal Intensive Care Unit, Institute for Maternal and Child Health, “IRCCS Burlo Garofolo”, 34137 Trieste, Italy; 26 Neonatology Unit, University Hospital of Udine, 33100 Udine, Italy; 27 ASST Pavia, Unità Operativa di Pediatria e Nido, Ospedale Civile, 27029 Vigevano, Italy; 28 Department of Infectious Diseases, Istituto Superiore di Sanità, 00161 Rome, Italy; 29 PhD Program in Clinical and Experimental Medicine, University of Modena and Reggio Emilia, 41121 Modena, Italy

**Keywords:** intrapartum antibiotic prophylaxis, early-onset sepsis, newborn, prevention, group B streptococcus, *Escherichia coli*

## Abstract

The effectiveness of “inadequate” intrapartum antibiotic prophylaxis (IAP administered < 4 h prior to delivery) in preventing early-onset sepsis (EOS) is debated. Italian prospective surveillance cohort data (2003–2022) were used to study the type and duration of IAP according to the timing of symptoms onset of group B streptococcus (GBS) and *E. coli* culture-confirmed EOS cases. IAP was defined “active” when the pathogen yielded in cultures was susceptible. We identified 263 EOS cases (GBS = 191; *E. coli* = 72). Among GBS EOS, 25% had received IAP (always active when beta-lactams were administered). Most IAP-exposed neonates with GBS were symptomatic at birth (67%) or remained asymptomatic (25%), regardless of IAP duration. Among *E. coli* EOS, 60% were IAP-exposed. However, IAP was active in only 8% of cases, and these newborns remained asymptomatic or presented with symptoms prior to 6 h of life. In contrast, most newborns exposed to an “inactive” IAP (52%) developed symptoms from 1 to >48 h of life. The key element to define IAP “adequate” seems the pathogen’s antimicrobial susceptibility rather than its duration. Newborns exposed to an active antimicrobial (as frequently occurs with GBS infections), who remain asymptomatic in the first 6 h of life, are likely uninfected. Because *E. coli* isolates are often unsusceptible to beta-lactam antibiotics, IAP-exposed neonates frequently develop symptoms of EOS after birth, up to 48 h of life and beyond.

## 1. Introduction

Early-onset sepsis (EOS) remains a leading cause of neonatal morbidity and death, particularly among neonates of the lowest gestational age [1]. Transmission occurs during delivery or shortly before, from a mother who is colonized at the genital site. Several neonatal and maternal risk factors (RFs) increase the likelihood of EOS in neonates born to *group B streptococcus* (GBS) colonized mothers. The RFs include GBS bacteriuria during the current pregnancy, previous infant with invasive GBS disease, membrane rupture lasting more than 18 h, intrapartum maternal temperature over 38 °C (a surrogate of chorioamnionitis), and preterm labor or membrane rupture before 37 weeks of gestation [2,3]. The investigation of RFs for GBS EOS has provided an important contribution to the development of effective preventive strategies targeting women at risk of transmitting GBS.

GBS and *Escherichia coli* (*E. coli*) are the most common organisms associated with EOS in high-income countries [4]. Strategies to prevent GBS EOS have been attempted since the 1970s. Despite imperfect study designs [5], three small, randomized clinical trials on intrapartum antibiotic prophylaxis (IAP) [6,7,8], suggested that administering antibiotics during labor and delivery was effective in preventing GBS EOS in neonates born to women at risk [6,9,10]. Large cohort studies have subsequently confirmed the great efficacy of IAP [3,11,12], which is currently a standard of care in many guidelines for preventing GBS EOS [3,13,14,15]. However, the current guidelines do not include specific recommendations for the prevention of *E. coli* EOS. The widespread use of IAP to reduce perinatal transmission of GBS has led to a substantial decrease in the incidence of overall EOS in high-income countries, to approximately 0.3–1 per 1000 live births (LBs) [16]. Intravenous penicillin remains the agent of choice for IAP, whereas intravenous ampicillin is an acceptable alternative. First-generation cephalosporins (i.e., cefazolin) are recommended for women allergic to penicillin with a low risk of anaphylaxis. In contrast, women with a high risk of anaphylaxis should be given clindamycin if the GBS isolate has been confirmed susceptible [15].

To maximize fetal exposure, U.S. guidelines recommend to give the loading dose of an appropriate antibiotic (such as penicillin, ampicillin, or cefazolin) [1,3,15,17], at least 4 h prior to delivery (“adequate IAP”). However, this threshold is not based on firm evidence and the optimal duration for preventing GBS EOS remains uncertain [18,19]. Furthermore, because of allergies to beta-lactams, up to ~5–10% of women may not receive appropriate antibiotics [20,21]. Finally, in many centers, the rates of women who receive ”adequate IAP” are often low (usually between 40% and 60%, rarely exceeding 70%) [19]. These findings explain the objective hindrance in achieving sufficient duration of IAP, In some guidelines inadequate IAP is still an indication to perform laboratory tests in asymptomatic neonates or to administer empirical antibiotics when tests are abnormal [3]. In a recent Italian survey, 76% of respondents reported laboratory evaluation and/or antimicrobial administration for asymptomatic, full-term neonates exposed to inadequate IAP [22]. Consequently, defining an optimal duration of IAP is essential to reduce unnecessary antibiotic use in neonates. Antibiotic exposure during the first postnatal week has remained disproportionately high, although the incidence of EOS in recent years has dramatically declined [23]. A large cohort study encompassing a total of 757,979 late-preterm and full-term neonates was carried out in Europe, North America, and Australia [23]. Antibiotic exposure during the first week of life ranged from 1.18% to 12.45% of neonates, although EOS was confirmed only in 1.5% among 14,139 infants treated with antibiotics. Noteworthy, 22% of these neonates were treated with antibiotics even though they remained asymptomatic. The identification of variables to classify newborns with high or low risk of EOS is urgently needed, to avoid unnecessary antibiotics.

The information provided by IAP exposure is useful for managing asymptomatic infants at risk of EOS, but is less relevant for infants who are already symptomatic at birth, since they undergo evaluation and empirical treatment, regardless of the duration of IAP [3,24].

We analyzed a large cohort of neonates exposed and unexposed to maternal IAP. The aim of the study was to assess whether non-beta-lactams or beta-lactam antibiotics given less or more than 4 h prior to delivery affect the time of onset of EOS symptoms as compared to unexposed neonates.

## 2. Materials and Methods

### 2.1. Study Design

A screening-based strategy (prenatal screening at 35–37 weeks of gestation) and IAP according to the CDC guidelines [3] are in place in Emilia-Romagna (a Northern region of Italy with approximately four million people and 35,000 live births (LBs)/year) [25,26,27]. A network of GBS active area-based surveillance has been launched in 2003. The network includes all regional birth facilities: eight microbiological laboratories, 10 level 1 centers (<1000 LBs/year; inborn criteria: ≥2000 g, ≥35 weeks), four level 2 centers (>1000 LBs/year; inborn criteria: ≥1500 g, ≥32 weeks), and eight neonatal intensive care units (no restrictions for in- and out-born neonates). GBS cases (positive blood or cerebrospinal fluid [CSF] culture) occurring in an infant younger than 3 months of age are notified to the coordinating center. To minimize missed cases, an e-mail is sent monthly to all regional consultant pediatricians and microbiological laboratories to ask for notification. Demographics, modes of delivery, RFs for EOS, and clinical information are obtained from the labor and delivery records by surveillance officers using a standardized form. Incomplete data is retrieved via a telephone call from the coordinating center.

Since 2016 a prospective surveillance of *E. coli* EOS has been added to that of GBS [28]; furthermore, 16 additional Italian centers outside the Emilia-Romagna region (in northern, central, and southern Italy) were included in the network. Although the vast majority of GBS and *E. coli* cases included in this study (200 out of 263, 76.0%) came from the Emilia-Romagna area-based surveillance (GBS from 2003 to 2021; *E. coli* from 2016 to 2021) this study has no epidemiologic purpose (i.e., incidence rates of GBS or *E. coli* EOS) and analyzes all GBS and *E. coli* EOS cases that have been entered in the network database, coming from intra- or extra-regional birthing centers.

Inclusion criteria were (i) GBS or *E. coli* positive blood and/or CSF culture collected within the first three postnatal days and (ii) neonates delivered between 2003 and 2021 (for GBS cases) or between 2016 and 2021 (for *E. coli* cases). No exclusion criteria were used, and infants of all gestational ages were enrolled.

Case reporting and isolate collection were determined to be an active surveillance of public health interest. The Ethical Committee of the coordinating centre (Azienda Ospedaliero-Universitaria Policlinico di Modena) approved the project (Prot. 910/2020).

To maintain patient confidentiality, spreadsheets submitted to the principal investigator were fully anonymous and did not include any identifiable data of patients or caregivers. Therefore, according to the policy of our ethics committee review board, patient consent was not required. The Strengthening the Reporting of Observational Studies in Epidemiology (STROBE) reporting guidelines were followed for this study.

### 2.2. Clinical and Microbiological Practices

According to the individual center policy, a single (1 mL) or double blood culture was obtained before antibiotic treatment from each neonate with clinically suspected EOS. Furthermore, some centers also collected blood culture in asymptomatic infants with maternal RFs for EOS. For each case of culture-proven EOS, maternal and neonatal information were collected from delivery and case medical records by using a standardized form. Maternal information included data regarding antenatal GBS screening, mode of delivery, IAP exposure, and RFs for EOS. Newborn data included the clinical presentation, infecting organism, antimicrobial susceptibility, and antibiotic treatment.

### 2.3. Definitions

- EOS case: yielding of GBS or *E. coli* in a blood or CSF fluid culture obtained within the first three postnatal days [29,30,31];
- Antenatal GBS screening: maternal vagino-rectal screening for GBS, performed within 5 weeks prior to delivery [3,32];
- Intrapartum antibiotic prophylaxis: intrapartum antibiotics administered i.v. for preventing GBS EOS;
- Adequate IAP: penicillin, ampicillin, or cefazolin administered at least 4 h prior to delivery;
- Active IAP: the pathogen yielded in cultures was susceptible to the antibiotic administered for IAP;
- Pre-term neonates: neonates born at <37 weeks’ gestation;
- Late preterm neonates: neonates born at 34–36 weeks’ gestation;
- Full term neonates: neonates born at ≥37 weeks’ gestation;
- Asymptomatic neonate: infant without any symptoms of EOS, with positive blood culture collected for maternal RFs.

### 2.4. Statistical Analysis

The analyses were performed using MedCalc version 9.3 (MedCalc Software, Ostend, Belgium). Continuous variables were expressed as mean ± standard deviation or median and range. The Student’s *t*-test and Levene’s test for assessing homoscedasticity or the Mann–Whitney rank sum test and χ^2^ or Fisher’s exact test were used to compare, respectively, the continuous and categorical variables between groups. The total number of live births in Emilia-Romagna was provided by the Regional Health Agency (728,106 from 2003 to 2022; of which 93% were full term and 7% were preterm). Furthermore, approximately 140,000 live births were delivered from 2016 to 2022 in extra-regional birthing centers.

## 3. Results

During the study period there were 868,106 live births, and the surveillance reported 263 cases of EOS, of which 191 were due to GBS and 72 were due to *E. coli*. Most infections (178 out of 263, 67.7%) were observed in full term neonates. Table 1 shows demographics, rates of symptomatic neonates and age at presentation in GBS and *E. coli* EOS. Median birth weight and weeks’ gestation were lower in neonates with *E. coli* as compared with those with GBS EOS; the rates of asymptomatic infants with GBS or *E. coli* EOS were similar, both in full-term and preterm infants.

Overall, 91 out of 263 (34.6%) neonates were exposed to IAP, of which 75.8% were exposed to beta-lactams. Neonates with *E. coli* EOS were more likely to be, IAP-, beta- and non-beta-lactams exposed (Table 2). GBS strains were more likely to be susceptible to an active IAP.

### 3.1. IAP-Exposure in GBS EOS and Timing of Symptoms

Among 191 GBS cases, 48 (25.1%) received intrapartum antibiotics. IAP-exposed full-term neonates developed symptoms significantly earlier than those unexposed, but no difference was found among preterm neonates whether IAP-exposed or unexposed. Symptoms were also significantly earlier in full-term infants who were exposed to antibiotics other than beta-lactams as compared to IAP-unexposed full-term neonates (Table 3).

**Figure 1:**
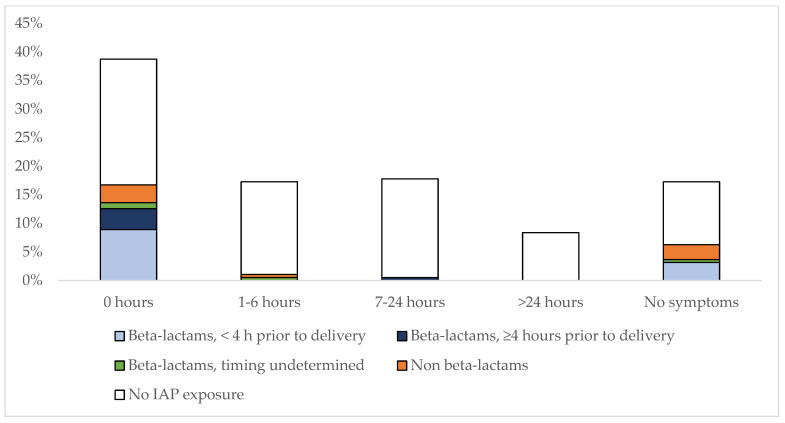
Hours of life at the onset of symptoms among 191 GBS cases according to IAP-exposure with different antibiotics. Bar graphs are presented as percentages. The number of cases for each different antibiotic and time interval is: beta-lactams, <4 h prior to delivery (0 h, n = 17; 7–24 h, n = 1; no symptoms, n = 6); beta-lactams, ≥4 h prior to delivery (0 h, n = 17; 7–24 h, n = 1); beta-lactams, timing undetermined (0 h, n = 2; 1–6 h, n = 1; no symptoms, n = 6); non beta-lactams (0 h, n = 6; 1–6 h, n = 2; no symptoms, n = 5); no IAP exposure (0 h, n = 42; 1–6 h, n = 31; 7–24 h, n = 33; >24 h, n = 16; no symptoms, n = 21).

Timing of onset of symptoms in the entire cohort of GBS cases according to IAP-exposure are shown in Figure 1. Among 48 GBS cases exposed to IAP, 12 (25%) remained asymptomatic; 32 (66.7%) were symptomatic at birth (of which 26 received beta-lactams: n = 17 inadequate IAP, N = 7 received adequate IAP, n = 2 unknown duration; 6 received non beta-lactams), and 4 (8.3%) developed symptoms after birth: 2 received beta-lactam IAP (the first newborn with unknown IAP duration and symptoms at 2 h of life; the second newborn with an adequate IAP and symptoms at 14 h). Two received non-beta-lactam IAP: 1 developed mild respiratory symptoms at 2 h life, and the second presented with septic shock at 4 h of life.

Eighty IAP-unexposed neonates developed symptoms after birth (up to 48 h of life and subsequently).

### 3.2. IAP-Exposure in E. coli EOS and Timing of Symptoms

Among 72 *E. coli* cases, approximately 60% were exposed to IAP, but a minority (8%) to an active IAP (Table 2). Age at the onset of symptoms did not differ in IAP-exposed versus unexposed neonates (Table 4).

**Figure 2:**
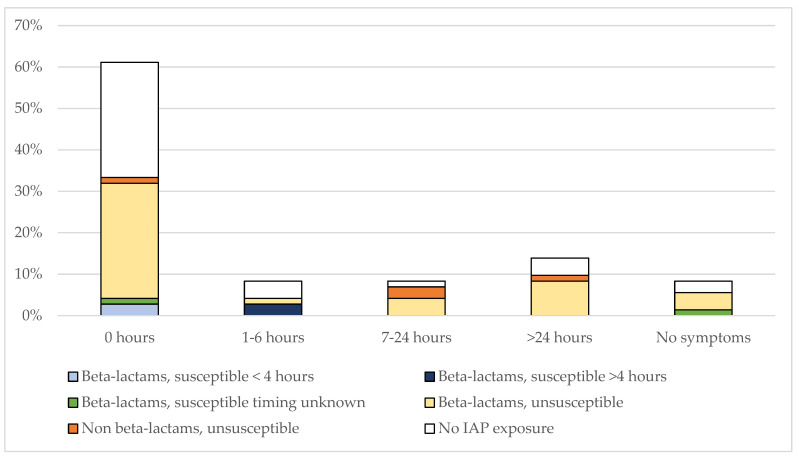
Hours of life at the onset of symptoms among 72 *E. coli* cases according to IAP-exposure with different antibiotics. Bar graphs are presented as percentages. The number of cases for each different antibiotic and time interval is: beta-lactams, susceptible, <4 h prior to delivery (0 h, n = 2); beta-lactams, susceptible, ≥4 h prior to delivery (1–6 h, n = 2); beta-lactams, susceptible, timing unknown (0 h, n = 1; no symptoms, n = 1); beta-lactams, unsusceptible (0 h, n = 20; 1–6 h, n = 1; 7–24 h, n = 3; >24 h, n = 6; no symptoms, n = 3); non beta-lactams, unsusceptible (0 h, n = 1; 7–24 h, n = 2; >24 h, n = 1); no IAP exposure (0 h, n = 20; 1–6 h, n = 3; 7–24 h, n = 1; >24 h, n = 3; no symptoms, n = 2).

The timing of EOS symptoms according to IAP exposure and different antibiotics administered is shown in Figure 2. Among 43 IAP-exposed neonates, 24 were symptomatic at birth; 15 neonates developed symptoms after birth (three infants between 1–6 h; five infants between 7–24 h, and seven beyond 24 h). Among six cases of EOS exposed to active IAP, three were symptomatic at birth (of which two received inadequate IAP, and one was unknown); two were symptomatic from 1 to 6 h of life (both received adequate IAP); and one remained asymptomatic (unknown duration of IAP). In contrast, most neonates who developed symptoms from 1 to >48 h of life (13/22, 60%) were exposed to inactive IAP; eight of them developed symptoms at ≥48 h of life.

Seven IAP-unexposed neonates developed symptoms after birth (up to 48 h of life and subsequently).

### 3.3. Comparison between GBS and E. coli EOS

Among all IAP-unexposed neonates, timing of symptoms was earlier in those with *E. coli* EOS. In contrast, IAP-exposed neonates with GBS EOS developed symptoms earlier than those with *E. coli* EOS (considering both the whole cohort and full-term neonates) (Table 5).

## 4. Discussion

Establishing an effective duration of IAP is important to manage asymptomatic neonates after birth; multivariate models for the management of at-risk for EOS infants have included information on timing and antibiotics administered [33]. This is the first study investigating the efficacy of IAP by correlating the timing of EOS symptom onset with the pathogen susceptibility to IAP. This information is essential to inform approaches in asymptomatic infants and avoid unnecessary antibiotics.

Our data show that the time of symptom onset is comparable in IAP-unexposed neonates with EOS due to GBS or *E. coli*; however, preterm neonates develop symptoms earlier than full-term neonates. The different pathogenesis of EOS may explain this finding; in fact, the pathogen causing EOS in preterm delivery is frequently transmitted to the fetus after maternal intra-amniotic infection, prior to the onset of labor [34]. In contrast, the pathogen causing EOS is acquired during labor (just before delivery) in full term neonates, or during the passage through the birth canal [1].

In the current study we found no clear cut-off duration for defining an “effective” IAP [35]. Rather, provided the loading dose had been fully administered, the key element was the pathogen’s antimicrobial susceptibility. IAP likely works by reducing the bacterial colony counts in the birth canal [36,37], and the absence of symptoms at birth in neonates exposed to an effective IAP confirms that they are uninfected, whatever the duration is.

Septic newborns exposed to an active IAP developed symptoms at birth or immediately after. In contrast, many IAP-unexposed neonates developed symptoms of EOS many hours after birth. This finding suggests an already established infection in utero, which cannot be prevented through IAP. Our data also showed an effect of non-beta-lactam antibiotics in preventing GBS infections. Indeed, in this cohort approximately half of IAP-exposed neonates (receiving intrapartum non-beta-lactam antibiotics) had a susceptible GBS isolate.

However, unlike GBS, most *E. coli* isolates were unsusceptible to beta-lactams. Given the growing relevance of *E. coli* in EOS [28] and the emerging problem of antimicrobial resistances [38], it is necessary to plan alternative strategies for preventing *E. coli* EOS. Like IAP-unexposed neonates, newborns with EOS due to unsusceptible *E. coli* developed symptoms at variable ages after birth, in many cases beyond 24 h of life, and as many as eight infants developed symptoms at ≥48 h of life. Our data strongly reinforce what we previously found for GBS and explain how IAP works [19,35,39,40].

These results can inform approaches for managing neonates. For clinicians who manage well-appearing newborns at risk for GBS EOS, it is crucial to be aware that virtually all beta-lactam-exposed newborns who remain asymptomatic after the first few hours of life will not develop GBS EOS. If a serial clinical observation approach is adopted, infants may be observed less frequently than previously suggested [41] and ideally, they could be discharged safely after the first 24 h of life. In contrast, infants delivered from a GBS negative (or unscreened) mother, with RFs for EOS, may still be at risk of EOS due to non-GBS pathogens, potentially resistant to beta-lactams. Consequently, their neonatal approach should be more cautious, and home discharge prior to 48 h of life would not be suggested.

This study has several limitations. First, we had incomplete information on the antimicrobial susceptibility of isolates, preventing a detailed analysis of all cases. However, information was missing for only a minority of cases. Moreover, data regarding non-beta-lactams in the prevention of GBS EOS are limited due to the small number of such cases we enrolled. Therefore, a firm conclusion regarding the protective efficacy of non-beta-lactam antibiotics cannot be drawn. A further limitation is inherent to the cohort study design. However, we recently detailed the difficulties in conducting randomized clinical trials on this topic and the several limitations and potential biases of previous non-randomized clinical studies [19]. Finally, we were unable to assess whether some neonates who developed symptoms at ≥48 h of life acquired the pathogen via vertical or (more likely) horizontal transmission; in fact, in some guidelines late-onset sepsis is defined as sepsis presenting after 48 h of life [42,43].

In conclusion, this large cohort study did not find any cut-off duration to define an “adequate” IAP. The appropriateness of IAP varies depending on the pathogen susceptibility to antibiotics administered. While beta-lactams are always effective against GBS, their activity against *E. coli* is often poor and cannot be defined a priori. Thus, neonates with *E. coli* infection have higher risk of developing symptoms of EOS later, up to 48 h of life and beyond.

## Figures and Tables

**Table 1:** Demographics, rates of symptomatic neonates and age at presentation in GBS and *E. coli* EOS cases.

	All Cases (n = 263)	GBS (n = 191)	*E. coli* (n = 72)	*p*
Median gestational age, wks (IQR)	38 (34–40)	39 (37–40)	34 (28.5–39)	<0.0001
Median birth weight, g (IQR)	3100 (2208.75–3500.00)	3220 (2776.25–3607.50)	2160 (1110.00–3127.50)	<0.0001
Preterm neonates, n (%)	85 (32.3)	42 (22.0)	43 (59.7)	<0.0001
Male sex, n (%)	134 (51)	97 (50.8)	37 (51.4)	0.9593
Symptomatic neonates, hours at presentation (IQR)	0.50 (0.00–8.00)	1.50 (0.00–4.00)	0.00 (0.00–0.00)	0.1004
All asymptomatic neonates (0–72 h of life), n (%)	39 (14.8)	33 (17.3)	6 (8.3)	0.1041
Asymptomatic full-term neonates, n (%)	38 (14.5)	32 (16.8)	6 (8.3)	0.1247
Asymptomatic preterm neonates, n (%)	1 (0.4)	1 (0.5)	0 (0)	0.6112

IQR, interquartile range.

**Table 2:** Comparison of GBS and E coli EOS according to IAP exposure.

	All Cases (n = 263)	GBS (n = 191)	*E. coli* (n = 72)	*p*
All IAP, n (%)	91 (34.6)	48 (25.1)	43 (59.7)	<0.0001
IAP with beta-lactams, n (%)	69 (26.2)	35 (18.3)	34 (47.2)	<0.0001
IAP with non beta-lactams, n (%)	22 (8.4)	13 (6.8)	9 (12.5)	<0.0001
Cases exposed to an active IAP, n (%)	47 (17.9)	41 (21.5)	6 (8.3)	0.0215
- Beta-lactams, n (%)	41 (15.6)	35 (18.3)	6 (8.3)	0.0717
- Non beta-lactams, n (%)	6 (2.3)	6 (3.1)	0 (0)	0.2899

IAP, intrapartum antibiotic prophylaxis (includes ampicillin, penicillin or cefazolin). All GBS cases were susceptible to beta-lactams.

**Table 3:** Timing of onset of symptoms according to IAP exposure in full-term and preterm neonates with GBS EOS.

	No IAP Administration (n = 122)	All IAP Administered (n = 36)	Non Beta-Lactams (n = 8)	*p*1	*p*2
Full-term neonates, median hours at the onset of symptoms (IQR)	6.00 (0.75–12.50)	0.00 (0.00–0.00)	0.00 (0.00–4.00)	<0.0001	0.0197
Preterm neonates, median hours at the onset of symptoms (IQR)	0.00 (0.00–2.75)	0.00 (0.00–0.00)	ND	0.0856	0.4213

IAP, intrapartum antibiotic prophylaxis; IQR, interquartile range; ND, not determined. *p*1, comparison between IAP not administered and all IAP administered. *p*2, comparison between IAP not administered and IAP administered with non-beta-lactam antibiotics.

**Table 4:** Timing of onset of symptoms according to IAP exposure in full-term and preterm neonates with *E. coli* EOS.

	No IAP Exposure (n = 10)	IAP Exposure (n = 8)	*p*
Full-term neonates, median hours at the onset of symptoms (IQR)	1.00 (0.00–6.00)	8.00 (0.00–45.00)	0.6965

IAP, intrapartum antibiotic prophylaxis.

**Table 5:** Hours of life at the onset of symptoms according to IAP exposure in full-term and preterm neonates with GBS and *E. coli* EOS.

	GBS (n = 158)	*E. coli* (n = 66)	*p*
All IAP-unexposed neonates, median hours (IQR)	4 (0.00–12.00)	0 (0.00–1.50)	0.0041
Full term IAP-unexposed neonates, median hours (IQR)	6 (4.00–8.00)	1 (0.00–56.22)	0.2143
Preterm IAP-unexposed neonates, median hours (IQR)	0 (0.00–2.75)	0 (0.00–0.00)	0.0957
All IAP-exposed neonates, median hours (IQR)	0 (0.00–0.00)	0 (0.00–5.58)	0.0267
Full term IAP-exposed neonates, median hours (IQR)	0 (0.00–0.00)	8 (0.00–45.00)	0.0296
Preterm IAP-exposed neonates, median hours (IQR)	0 (0.00–2.00)	0 (0.00–0.00)	0.0957

## Data Availability

The data that support the findings of this study are openly available (attachment to this submission).
